# Survival outcomes of neoadjuvant versus adjuvant chemotherapy in triple-negative breast cancer: a meta-analysis of 36,480 cases

**DOI:** 10.1186/s12957-020-01907-7

**Published:** 2020-06-15

**Authors:** Lin-Yu Xia, Qing-Lin Hu, Jing Zhang, Wei-Yun Xu, Xiao-Shi Li

**Affiliations:** 1 Department of Thyroid and Breast Surgery, The First Affiliated Hospital of Chengdu Medical College, 278 Baoguang Avenue Middle Section, Xindu District, Chengdu City, 610500 Sichuan Province China; 2 Department of Breast Surgery, Mianyang Central Hospital, Mianyang, Sichuan China

**Keywords:** Triple-negative breast cancer, Neoadjuvant, Adjuvant chemotherapy, Survival outcomes, Meta-analysis

## Abstract

**Background:**

The survival outcomes of neoadjuvant chemotherapy (NACT) versus adjuvant chemotherapy (ACT) for patients with triple-negative breast cancer (TNBC) remain unclear. Therefore, in this study, a meta-analysis was conducted to analyze current evidence on the survival outcomes of NACT versus ACT in TNBC.

**Methods:**

A systematic search was performed on the PubMed and Embase databases to identify relevant articles investigating the survival outcomes of NACT versus ACT in TNBC.

**Results:**

A total of nine studies involving 36,480 patients met the selection criteria. Among them, 10,728 (29.41%) received NACT, and 25,752 (70.59%) received ACT. The pathological complete response (pCR) rate was 35% (95% CI = 0.23–0.48). Compared with ACT, the overall survival (OS) of NACT was poor (HR = 1.59; 95% CI = 1.25–2.02; *P =* 0.0001), and there was no significant difference in disease-free survival (DFS) between the two treatments (HR = 0.85; 95% CI = 0.54–1.34; *P* = 0.49). NACT with pCR significantly improved the OS (HR = 0.53; 95% CI = 0.29–0.98; *P* = 0.04) and DFS (HR = 0.52; 95% CI = 0.29–0.94; *P* = 0.03), while the OS (HR = 1.18; 95% CI = 1.09–1.28; *P* < 0.0001) and DFS (HR = 2.36; 95% CI = 1.42–3.89; *P* = 0.0008) of patients with residual disease (RD) following NACT were worse compared to those receiving ACT.

**Conclusion:**

These findings suggest that, for TNBC, NACT with pCR is superior to ACT in improving OS and DFS, and it turns to be opposite when patients are receiving NACT with RD.

## Introduction

Breast cancer is the most common cancer in women. Globally, nearly 1.2 million to 1.4 million women are diagnosed with breast cancer, and about 400,000 die of breast cancer [1]. TNBC is defined as a type of breast cancer lacking the three most common types of receptors namely, estrogen receptor (ER), progesterone receptor (PR), and HER-2 expression which are known to drive the growth of breast cancer. TNBC accounts for 12–20% of all breast cancers, and it is characterized by high pathological grade, strong invasiveness, local recurrence, high metastasis rate, and poor prognosis [2–4]. Therefore, systemic treatment should be administered in the early stage after diagnosis. In the past, ACT has been the standard treatment for TNBC, but now more and more patients with TNBC have adopted NACT because it can control systemic micrometastases, reduce the tumor burden, provide surgical or conservative breast surgery opportunities for locally advanced breast cancer patients, and allow detection of tumor sensitivity to chemotherapeutic drugs. pCR after NACT improves tumor-free survival rate of patients [5, 6]. Studies have shown that the rate of pCR in patients with TNBC receiving NACT is significantly higher than that of non-TNBC patients [7–11]. This indicates that NACT is effective for TNBC patients. Currently, studies have compared the prognosis of NACT and ACT in patients with TNBC, but the results are contradictory [12–14]. Thus, whether NACT yields better survival outcomes in TNBC than ACT is still controversial. Our study aimed to compare the survival outcomes of NACT versus ACT in TNBC by meta-analysis.

## Material and methods

### Search strategy

A systematic literature search was performed on the PubMed and Embase databases for the period up to January 18, 2020, to identify eligible studies. The keywords used in the search strategy were triple-negative breast neoplasms OR triple-negative breast cancer OR triple-negative breast carcinoma AND neoadjuvant OR preoperative AND Adjuvant chemotherapy OR chemotherapy. A total of nine articles with a total of 36,480 patients met the eligibility criteria [12–20]. The inclusion of studies was not limited to geographical location of study or publication language.

### Inclusion and exclusion criteria

**Fig. 1:**
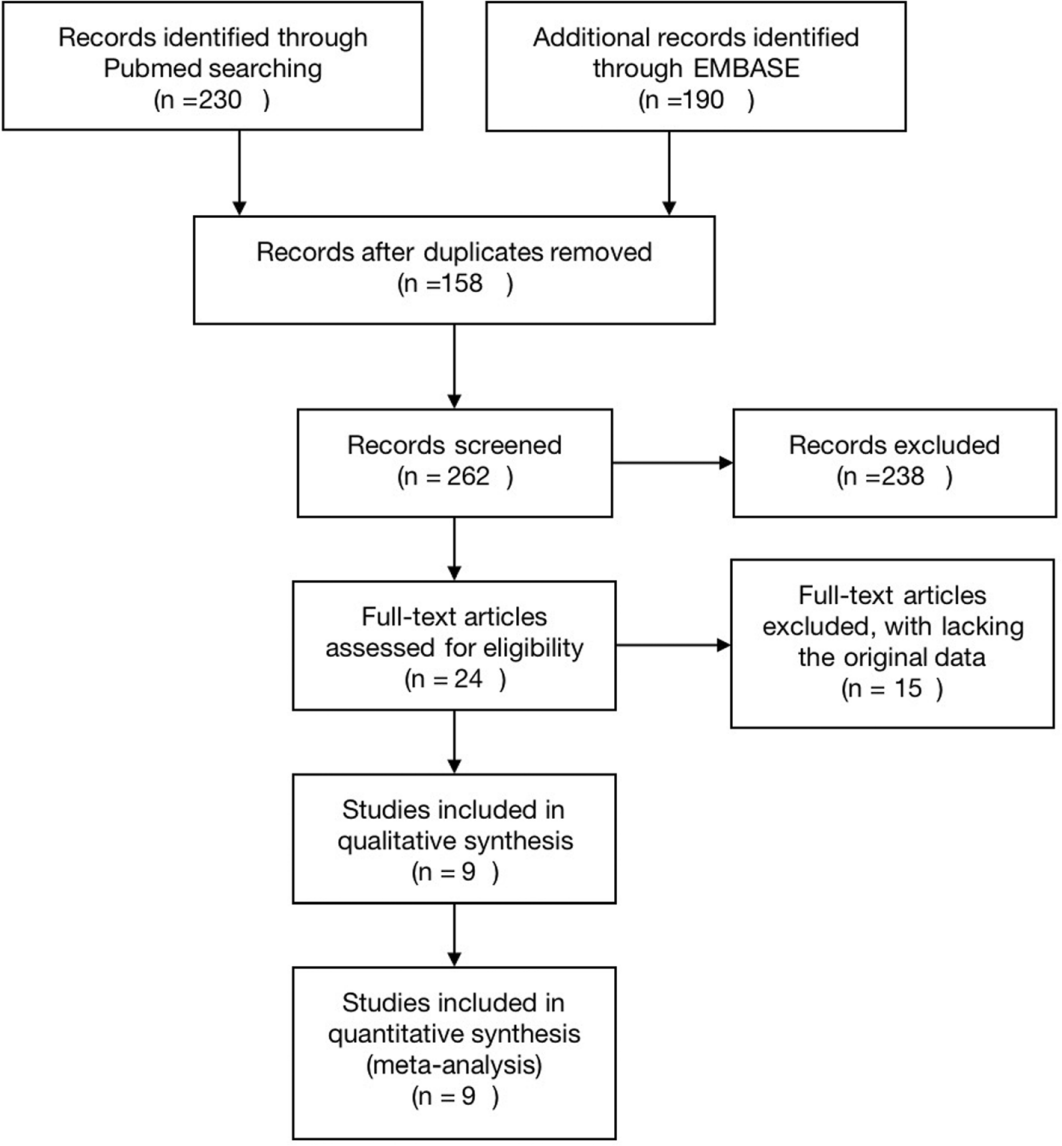
Flowchart explaining the article selection

Eligible studies met the following inclusion criteria to ensure only high-quality studies were considered for this analysis. Inclusion criteria are as follows: (1) patients diagnosed with TNBC, (2) the study compared the survival outcomes of NACT with ACT, (3) the study assessed the overall prognosis of TNBC, (4) the study reported survival outcomes in terms of OS and/or DFS. The exclusion criteria were as follows: (1) articles lacking the original data, (2) studies lacking information on survival outcomes in TNBC, (3) articles not reporting or giving an estimate of the hazard ratio (HR) and a 95% confidence interval (95% CI). Figure 1 illustrates the eligibility criteria of articles enrolled in this meta-analysis.

### Data extraction and quality assessment

A standardized data extraction form was used to extract relevant information from each study. For each eligible study, the following information was extracted: the first author’s name, publication year, patients’ countries, patients’ ages, study design, number of participations, tumor characteristics, chemotherapy regimen, and follow-up results.

The Newcastle–Ottawa quality assessment scale was used to assess the quality of each included study [21]. The NOS evaluated non-randomized studies based on three criteria: patient selection (four stars), study group comparability (two stars), and outcome evaluation (three stars). Only studies with a score of 6 or above were included in the meta-analysis (See Supplementary Table 1, Additional File 1).

### Statistical analysis

Meta-analysis was conducted using RevMan version 5.3 (RevMan, version 5.3 for Windows; Cochrane Collaboration, Oxford, UK). The hazard ratios (HRs) with 95% CIs were calculated to estimate the association between the DFS and OS of NACT and ACT in TNBC. Published data and Kaplan-Meier survival curves were used to extract the HR estimates according to the methods reported by Tierney et al. if the HRs were not directly provided [22]. Chi-squared based Q-test (*P* > 0.10) and *I*^2^ were used to determine statistical heterogeneity within the studies. When *I*^2^ < 50%, the studies were considered to have acceptable heterogeneity, and the fixed-effects model was used. Otherwise, the random-effects model was used. All *P* values were two-sided, and *P* < 0.05 was considered to be statistically significant. Publication bias was assessed using funnel’s plot and quantified by Egger’s test [23]. These analyses were performed using Stata 15.0 (StataCorp, College Station, Tex).

## Results

Figure 1 shows the process of selecting the included studies. A total of 420 articles were first identified for evaluation. Among these, 158 were excluded because they were duplicates while 238 were irrelevant to this study. Based on the inclusion and exclusion criteria described above, 15 were excluded from further analysis. Therefore, 9 publications were eligible for the meta-analysis. Among the 9 studies, 7 were retrospective while 2 were prospective. Tables 1 and 2 present the characteristics of the included studies.

**Table 1:** Main characteristics and results of the eligible studies

First author	Year	Country	Study type	*N*	Clinical stage	Chemotherapy regimen	pCR rate	Follow-up (median) (year)	HR estimation	Outcomes
Clifton	2018	USA	Prospective	319	I–II	A/T	0.538	6.33	Survival curve	OS, DFS
Fisher	2012	USA	Retrospective	385	I–III	NA	0.17	2.5	Survival curve	OS
Kennedy	2010	USA	Retrospective	405	I–III	NA	NR	4.3	Survival curve	OS
Sharma	2015	USA	Retrospective	146	I–II	A/T	NR	3.08	Given by author	DFS
Cheng	2017	NR	Retrospective	15,483	I–III	NA	NR	2	Given by author	OS
Yang	2018	China	Prospective	67	II–III	A/T	0.194	6.5	Given by author	OS
Biswas	2017	USA	Retrospective	420	I–III	A/T	0.33	3.9	Survival curve	OS
Bagegni	2019	USA	Retrospective	19,151	II-III	NA	0.474	2.5	Survival curve	OS
Philipovskiy	2019	USA	Retrospective	104	I–III	A/T	0.4	6	Given by author	OS,DFS

*NR* not reported, *A/T* adriamycin/taxane, *OS* overall survival, *DFS* disease-free survival

**Table 2:** Patient and tumor characteristics in the neoadjuvant and adjuvant groups from the studies

First author	*N* NACT/ACT	Median age (year)	Clinical stage		Nuclear grade		Histology		Local treatment	
		NACT/ACT	NACT	ACT	NACT	ACT	NACT	ACT		
			I/II/III	I/II/III	1/2/3	1/2/3	IDC/ILC/Other	IDC/ILC/Other	M	B
Clifton	132/187	< 50, 102/144	15/70/0	20/65/0	0/4/81	2/1/79	84/0/1	83/0/2	162	157
		≥ 50, 30/43								
Fisher	151/234	< 50, 82/96	10/85/49	81/91/11	2/15/130	2/25/200	120/7/24	190/7/37	NR	NR
		≥ 50, 69/138								
Kennedy	154/251	50/53	3/80/43	89/101/19	0/14/135	5/34/197	130/14/10	198/28/25	207	198
Sharma	67/79	52/58	NR	NR	NR	NR	NR	NR	NR	NR
Cheng	4335/11,148	< 50, 1951/3456	NR/NR/1517	NR/NR/669	NA	NA	NR	NR	NR	NR
		≥ 50, 2384/7692								
Yang	36/31	NA	NA	NA	NR	NR	NA	NA	NR	NR
Biswas	202/218	51/51	1/105/96	69/117/32	NA	NA	NR	NR	NA	NA
Bagegni	5621/13,530	51.9/55.7	0/3843/1778	0/12142/13,88	26/649/4530	102/1328/11,391	NR	NR	NR	NR
Philipovskiy	30/74	50.4/53	3/11/16	16/41/17	NR	NR	100/0/4^a^		45	54

*IDC* invasive ductal carcinoma, *ILC* invasive lobular carcinoma, *NR* not reported, *NA* not replied, *M* mastectomy, *B* breast conserving surgery

^a^The data is the sum of neoadjuvant and adjuvant groups

**Fig. 2:**
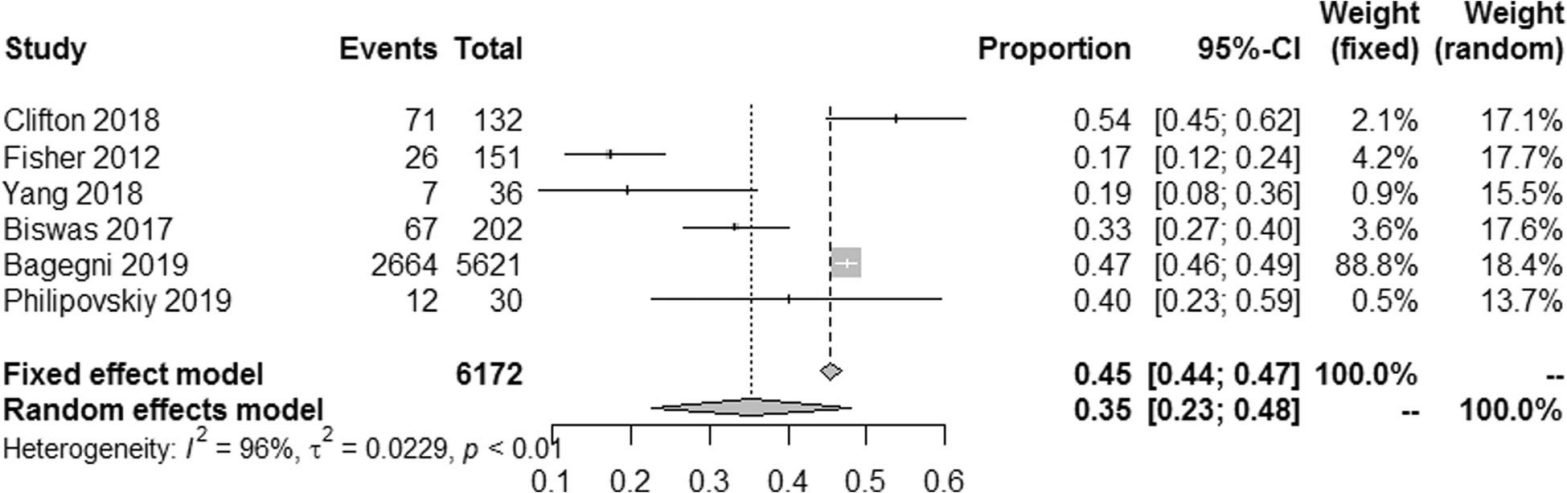
pCR rate ratios of NACT in TNBC patients

**Fig. 3:**
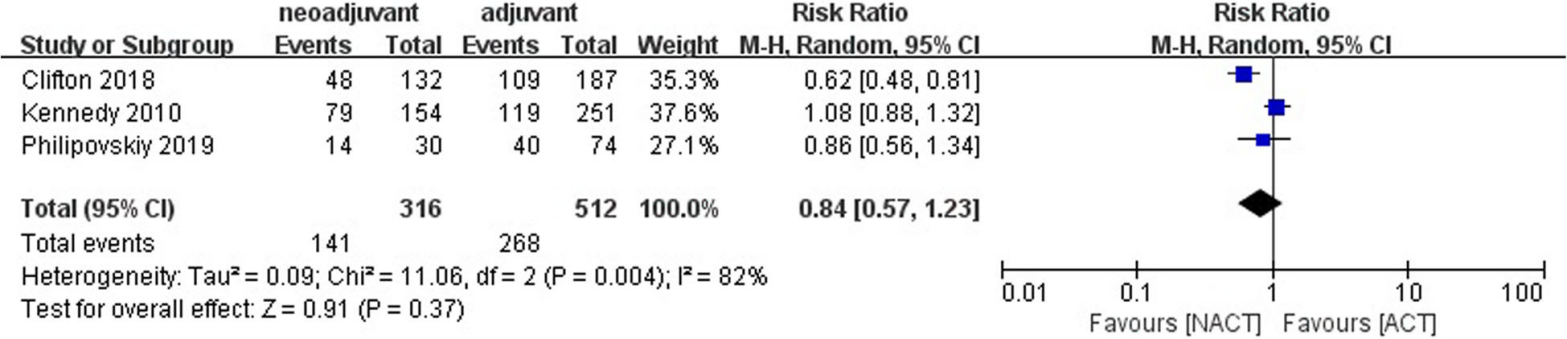
Forest plot of the RR for the breast conserving surgery rate for NACT vs. ACT group in TNBC patients

Among the 36,480 patients included, 10,728 (29.41%) received NACT, and 25,752 (70.59%) received ACT. A pCR rate of 35% (95% CI = 0.23–0.48) was obtained in 6172 patients receiving NACT from six studies [12, 13, 17–20] (Fig. 2), and heterogeneity was detected in these data (*I*^2^ = 96%, *P* < 0.01). Three studies reported the breast conserving surgery rate, from which we found that the breast conserving rate after NACT was lower than ACT (RR = 0.84; 95% CI = 0.57–1.23; *P* = 0.37) [12, 14, 20] (Fig. 3), and heterogeneity was detected in these data (*I*^2^ = 82%, *P* = 0.004).

**Fig. 4:**
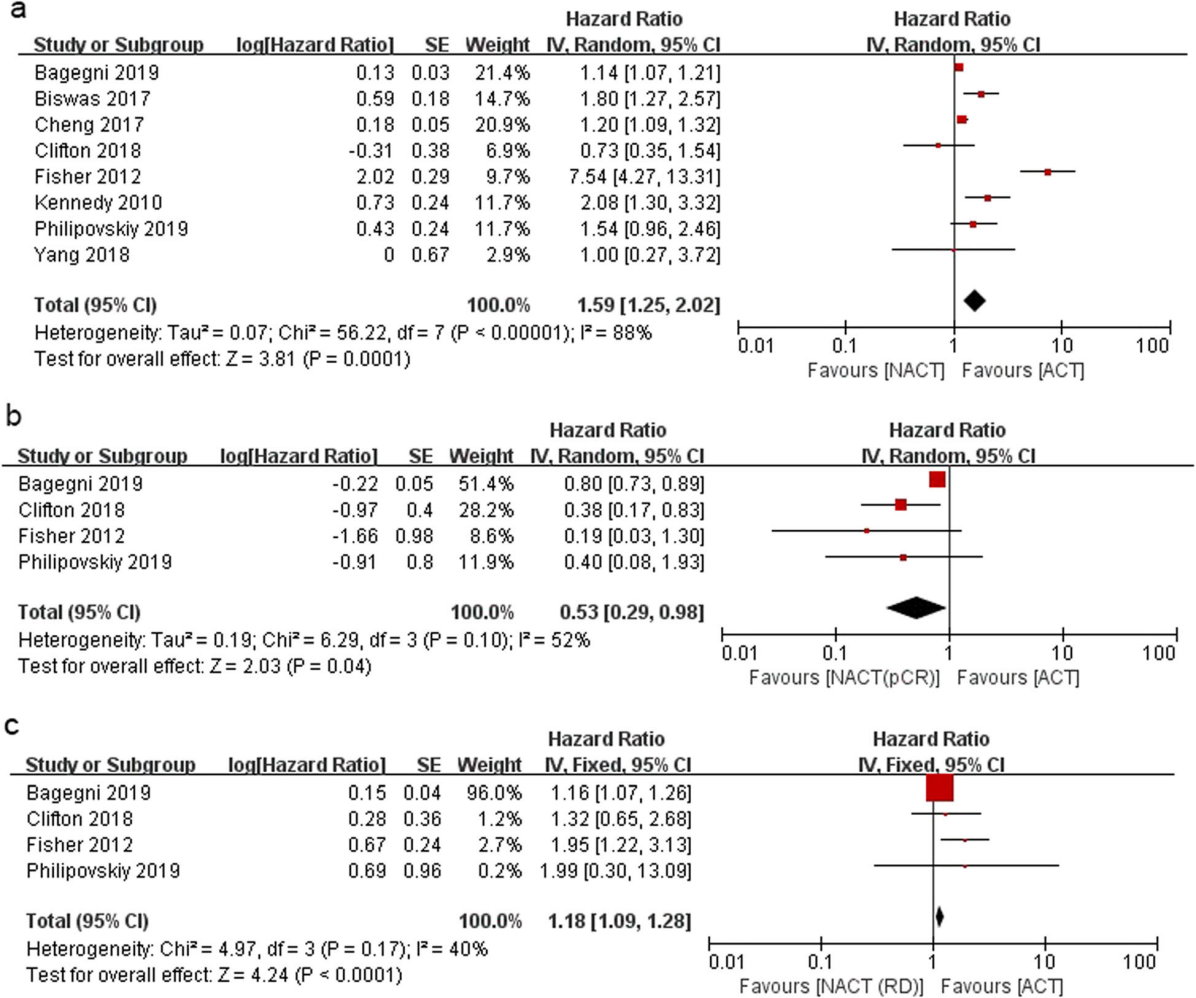
Forest plot of the HR for OS in TNBC patients for NACT vs. ACT (**a**), NACT with pCR vs. ACT (**b**), and NACT with RD vs. ACT (**c**)

The OS of NACT versus ACT in TNBC in the entire study was evaluated in the Clifton, Fisher, Kennedy, Cheng, Yang, Biswas, Bagegni, and Philipovskiy trials with a sample size of 36,422 [12–14, 16–20]. After a median follow-up of 4.12 years, NACT led to worse OS than ACT with an HR of 1.59; 95% CI (1.25–2.02); *P =* 0.0001. Significant heterogeneity existed among the studies (*I*^2^ = 88%, *P* < 0.000001). Unlike TNBC patients who received ACT, those with RD who were put on NACT had worse OS (HR = 1.18; 95% CI = 1.09–1.28; *P* < 0.0001), while those who achieved pCR following NACT had significant OS benefits (HR = 0.53; 95% CI = 0.29–0.98; *P* = 0.04) in the studies of Clifton, Fisher, Bagegni, and Philipovskiy [12, 13, 19, 20]. Heterogeneity did not exist among the included studies as shown in Fig. 4.

**Fig. 5:**
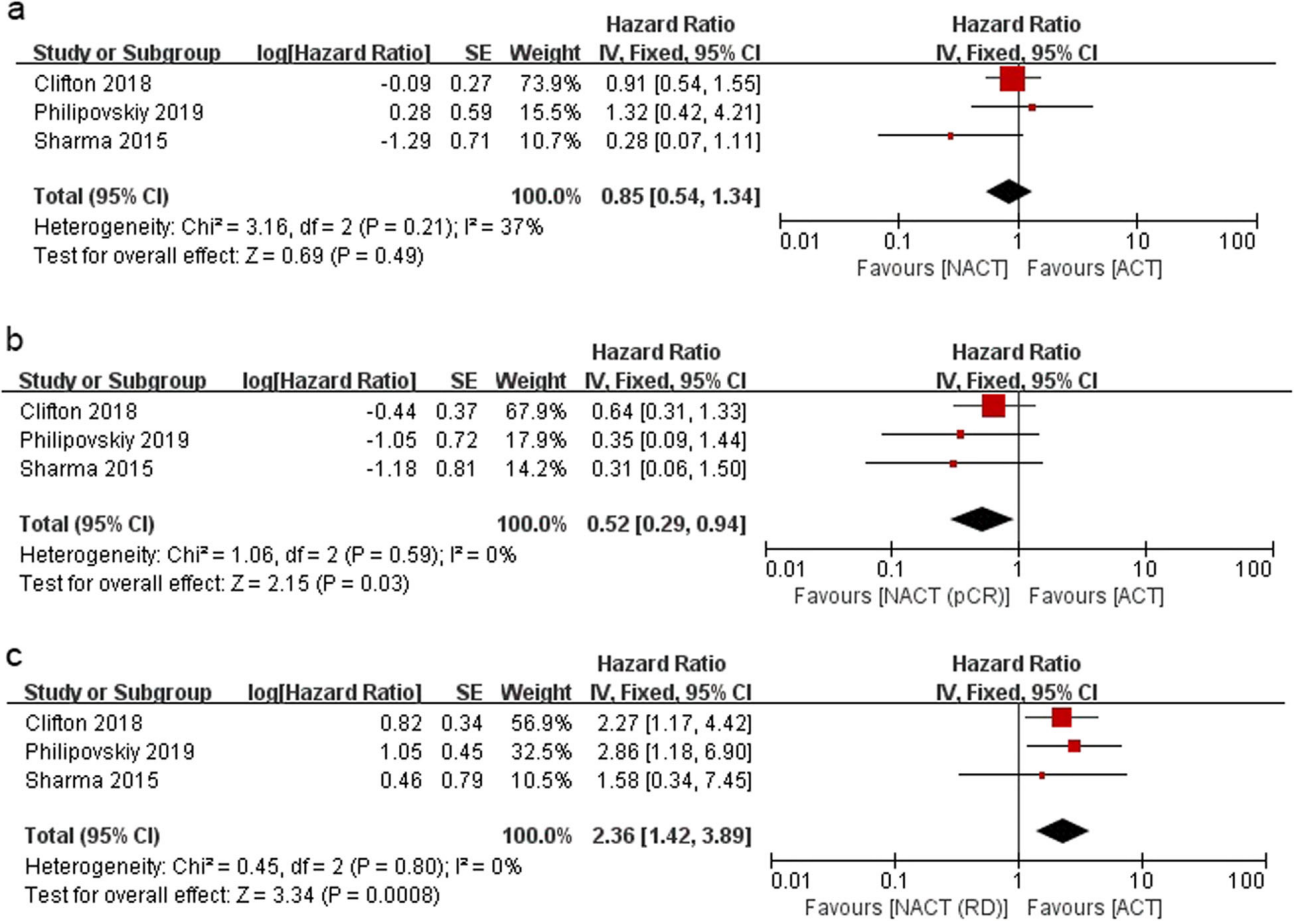
Forest plot of the HR for DFS in TNBC patients for NACT vs. ACT (**a**), NACT with pCR vs. ACT (**b**) and NACT with RD vs. ACT (**c**)

The trials by Clifton, Sharma, and Philipovskiy [12, 15, 20] reported DFS after NACT versus ACT in TNBC. A total of 569 patients were included in the pooled analysis with a median follow-up time of 5.14 years. There was no statistically significant difference between NACT and ACT arms on the DFS (HR = 0.85; 95% CI = 0.54–1.34; *P* = 0.49). Compared with patients receiving ACT, those who underwent NACT with pCR had a better DFS (HR = 0.52; 95% CI = 0.29–0.94; *P* = 0.03), and those with RD had a worse DFS (HR = 2.36; 95% CI = 1.42–3.89; *P* = 0.0008). Heterogeneity did not exist among the included studies as shown in Fig. 5.

Publication bias was detected by funnel plots and Egger’s test (See Supplementary Figure 1, Additional File 2). All *P* values were > 0.05 (See Supplementary Table 2, Additional File 3), indicating no potential publication bias.

## Discussion

TNBC is an aggressive form of breast cancer which is highly invasive and prone to recurrence and metastasis. We analyzed 36,480 patients in 9 studies and concluded that for TNBC patients, the OS of ACT exceeds that of NACT but there was no significant difference in DFS between the two treatments. Compared with ACT, patients with RD following NACT had worse OS and DFS, while those who achieved pCR had a better OS and DFS.

Three large randomized trials estimating NACT and ACT, NSABP B-18, EORTC 10902, and IBBGS, found that there was no significant difference in survival between NACT and ACT [24–26]. However, these trials did not differentiate breast cancer molecular subtypes. The results of our study only apply to the TNBC subtype of breast cancer. In our analysis, ACT was superior to NACT in improving the survival outcomes. This may be related to the fact that patients with severe disease were more likely to receive NACT. Studies have shown that patients receiving NACT have larger tumors, nodal positivity, and advanced clinical stage compared to those receiving ACT [13, 14, 16]. Although they try to control these factors that may affect the survival results in the multivariate model, we did not exclude some factors that are not included in the model which can potentially interfere with the choice of NACT or ACT. So the worse outcome of NACT may be partially caused by the biology of disease, so it is still noteworthy. Another reason for the lower OS in patients with TNBC who received NACT is the “delay effect” [14]. TNBC is characterized by increased risk of bone and central nervous system metastasis [27, 28]. NACT does not kill tumors when used for the first time as surgery does. Hence, patients receiving NACT may develop axillary metastases. Therefore, the observed survival benefit of ACT in our study may be a result of earlier tumor debulking, decreased opportunity for systemic tumor seeding, and systemic micro-metastases.

Consistent with our study, other studies have confirmed that patients with TNBC have a better prognosis after achieving pCR following NACT [7, 29]. Although patients who received NACT may have advanced disease, achieving pCR following NACT significantly improved survival. This suggests that in our study, compared with all patients receiving NACT, the survival advantage of ACT is determined by the residual disease after NACT. In our meta-analysis, the pCR rate is 35% (95% CI = 0.23–0.48; *P* < 0.01) (Fig. 2). In these studies, patients with early stage, small tumor and negative lymph node were more likely to achieve pCR. All studies reported a pCR rate below 50%, except that of Clifton where the pCR rate was 54%. Therefore, the high rate of RD is associated with a poor survival rate of NACT.

In this meta-analysis, the DFS was not significantly different between NACT and ACT arms. This differs from other studies. A study involving 4756 breast cancer patients showed that women who received NACT had higher local recurrence rates within 15 years (21.4% vs 15.9%) than those who received ACT (RR 1.37; 95% CI = 1.17–1.61; *P* = 0.0001) [30]. In the 4756 breast cancer patients, the risk increased significantly after 0–4 years (RR 1.35; 95% CI = 1.11–1.64) and 5–9 years (RR = 1.53; 95% CI = 1.08–2.17). Women who received NACT in their study were more likely to take breast-conserving treatments than those who received ACT (65% vs 49%). Breast-conserving surgery after NACT may increase the risk of local recurrence. Mauri et al. validated this through a meta-analysis of 9 randomized trials involving 3946 patients. They found that the risk of local recurrence of NACT group was significantly higher than that of ACT group due to the higher breast-conserving surgery rate in NACT cohort (RR = 1.22; 95% CI = 1.04–1.43; *P* = 0.018) [31]. Local recurrence following breast-conserving surgery may be caused by the disunity of tumor regression model after NACT, the difficulty of locating tumors, and breast-conserving surgery [32, 33]. By contrast, there was no significant difference in breast-conserving surgery rate between NACT and ACT (RR = 0.84; 95% CI = 0.57–1.23; *P* = 0.37) (Fig. 3) in this meta-analysis. This can be used to explain the discrepancies between our results and those reported in other studies.

This meta-analysis has some limitations. One of the limitations is that 8 studies analyzed the overall survival benefits of TNM stage I–III patients without distinguishing the early and late stages of the disease. Hence, we did not compare the survival benefits according to different stages of the disease. As proved in this study, TNBC with negative lymph node, small tumor, and early stage are more likely to achieve pCR in NACT. The poor survival benefit of NACT compared with ACT is determined by patients with RD. If all patients were in the early stage of disease, NACT may yield a higher pCR rate and a better survival benefit than ACT. Despite this limitation, we conclude that NACT with pCR can significantly improve survival in TNBC. In addition, the HR and 95% CI extracted from the survival curves may be less reliable than those directly obtained from the articles. Finally, 7 studies included in our analysis were retrospective researches, and they probably have potential biases.

## Conclusion

This meta-analysis shows that ACT results in a better OS for TNBC patients than NACT. However, there is no significant difference in DFS between the two treatments. Notably, NACT improves OS and DFS in patients achieving pCR. Thus, NACT may be more effective in patients predicted to achieve pCR, while ACT is suitable for patients who cannot achieve pCR. We recommend that well-designed trials be conducted to confirm our results.

## Supplementary information

**Additional file 1: Supplementary Table 1.** Risk of bias in the included cohort studies (by the Newcastle–Ottawa quality assessment tool)

**Additional file 2: Supplementary Figure 1.** Funnel plot of the HR for OS in TNBC patients for NACT vs. ACT (a), NACT with pCR vs. ACT(b) ,NACT with RD vs. ACT(c), for DFS in TNBC patients for NACT vs. ACT (d), NACT with pCR vs. ACT(e) and NACT with RD vs. ACT(f)

**Additional file 3: Supplementary Table 2.** Results of Egger’s tests for publication bias

## Data Availability

All the data are available without restriction. Researchers can obtain data from the corresponding author.

## Abbreviations

NACT: Neoadjuvant chemotherapy
ACT: Adjuvant chemotherapy
TNBC: Triple-negative breast cancer
pCR: Pathological complete response
OS: Overall survival
DFS: Disease-free survival
RD: Residual disease
