# Supplementary material for: Survival outcomes of neoadjuvant versus adjuvant chemotherapy in triple-negative breast cancer: a meta-analysis of 36,480 cases
Source: World J Surg Oncol. 2020 Jun 15;18:129. doi: 10.1186/s12957-020-01907-7 (PMC7296918; doi:10.1186/s12957-020-01907-7)
Supplement: Supplementary file 1 — Additional file 1: Supplementary Table 1. Risk of bias in the included cohort studies (by the Newcastle–Ottawa quality assessment tool) [file 12957_2020_1907_MOESM1_ESM.docx]

Supplementary Table 1 Risk of bias in the included cohort studies (by the Newcastle–Ottawa quality assessment tool)

|  | **Representativeness of the exposed cohort^a^** | **Selection of the non-exposed cohort**^a^ | **Ascertainment of exposure**^a^ | **Demonstration that outcome of interest was not present at start of study**^a^ | **Comparability of cohorts on the basis of the design or analysis^b^** | **Assessment of outcome**^a^ | **Was follow-up long enough for outcome to occur**^a^ | **Adequacy of follow-up of cohorts**^a^ | **Total quality scores** |  |
| --- | --- | --- | --- | --- | --- | --- | --- | --- | --- | --- |
| **Clifton 2018** | * | * | * | * | * | * | * | * | 8 |  |
| **Fisher 2012** | * | * | * | * | * | * | * | * | 8 |  |
| **Kennedy 2010** | ***** | * | * | * | * | * | * | * | 8 |  |
| **Sharma 2015** | ***** | * | * | * | * | * | * | * | 8 |  |
| **Cheng 2017** | ***** | * | * | * | * | * | - | - | 6 |  |
| **Yang 2018** | - | - | * | * | * | * | * | * | 6 |  |
| **Biswas 2017** | ***** | * | * | * | * | * | * | * | 8 |  |
| **Bagegni 2019** | * | * | * | * | ** | * | * | * | 9 |  |
| **Philipovskiy 2019** | **-** | * | * | * | * | * | * | * | 7 |  |

a A study can be awarded a maximum of one star for each numbered item.

b A maximum of two stars can be awarded for Control for important factor or additional factor.
