# Supplementary figures and images for: Survival outcomes of neoadjuvant versus adjuvant chemotherapy in triple-negative breast cancer: a meta-analysis of 36,480 cases
Source: World J Surg Oncol. 2020 Jun 15;18:129. doi: 10.1186/s12957-020-01907-7 (PMC7296918; doi:10.1186/s12957-020-01907-7)

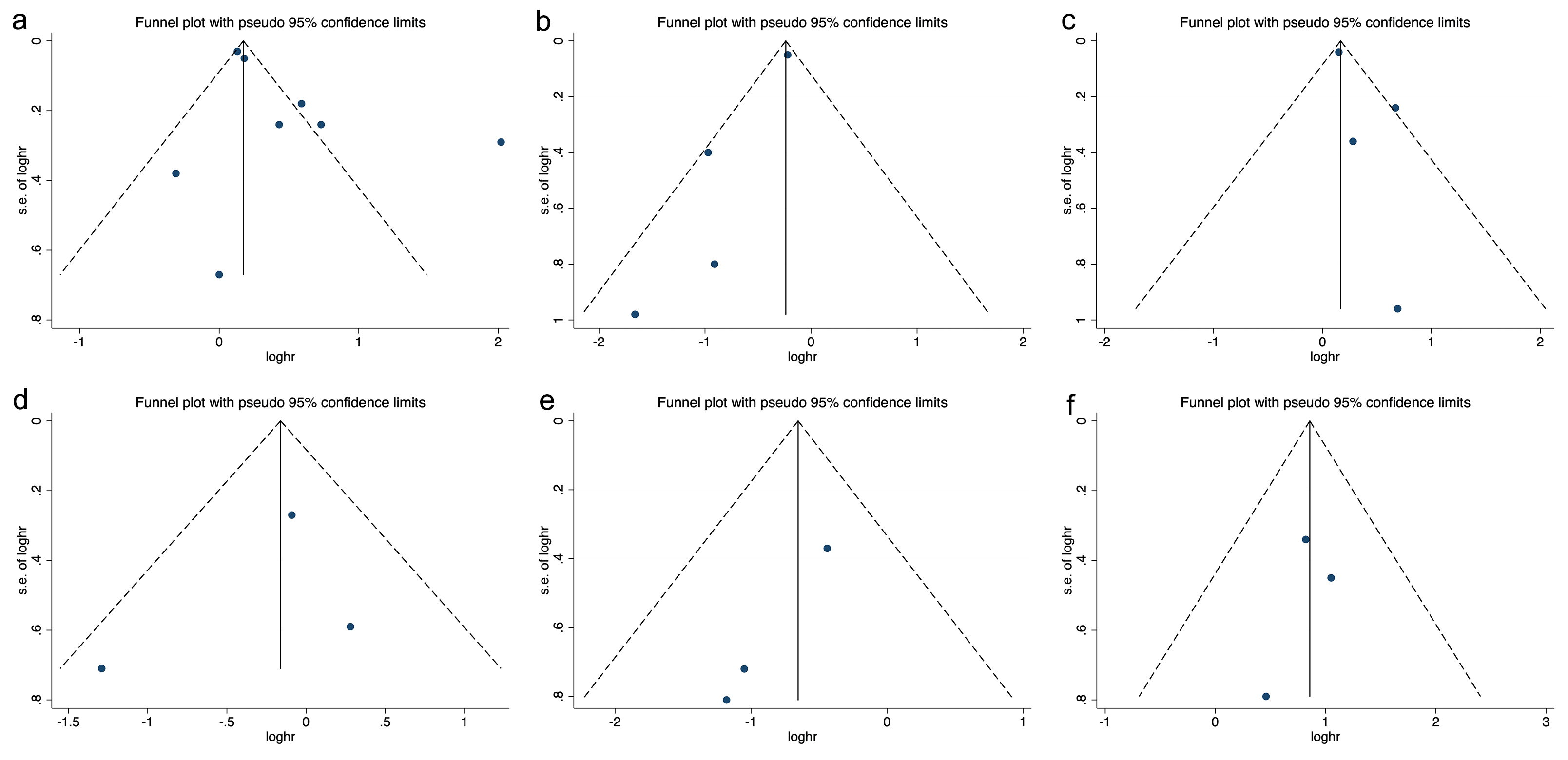

Supplement: Supplementary file 2 — Additional file 2: Supplementary Figure 1. Funnel plot of the HR for OS in TNBC patients for NACT vs. ACT (a), NACT with pCR vs. ACT(b) ,NACT with RD vs. ACT(c), for DFS in TNBC patients for NACT vs. ACT (d), NACT with pCR vs. ACT(e) and NACT with RD vs. ACT(f) [file 12957_2020_1907_MOESM2_ESM.jpg]
