# Supplementary material for: Survival outcomes of neoadjuvant versus adjuvant chemotherapy in triple-negative breast cancer: a meta-analysis of 36,480 cases
Source: World J Surg Oncol. 2020 Jun 15;18:129. doi: 10.1186/s12957-020-01907-7 (PMC7296918; doi:10.1186/s12957-020-01907-7)
Supplement: Supplementary file 3 — Additional file 3: Supplementary Table 2. Results of Egger’s tests for publication bias [file 12957_2020_1907_MOESM3_ESM.docx]

Supplementary Table 2 Results of Egger’s tests for publication bias

| End point | *t* | *P* |  |  |  |  |
| --- | --- | --- | --- | --- | --- | --- |
| OS(NACT VS ACT) | 1.75 | 0.131 |  |  |  |  |
| OS(NACT(pCR) VS ACT) | -4.31 | 0.051 |  |  |  |  |
| OS(NACT(RD) VS ACT) | 1.61 | 0.25 |  |  |  |  |
| DFS(NACT VS ACT) | -0.52 | 0.696 |  |  |  |  |
| DFS(NACT(pCR) VS ACT) | 9.89 | 0.06 |  |  |  |  |
| DFS(NACT(RD) VS ACT) | -0.60 | 0.658 |  |  |  |  |
